# Re-evaluating gut microbiome signatures of post-antibiotic dietary fiber intake in a large adult cohort

**DOI:** 10.1186/s13104-026-07708-7

**Published:** 2026-02-14

**Authors:** Yuwei Tang, Xi Fu, Yu Sun

**Affiliations:** 1 State Key Laboratory of Swine and Poultry Breeding Industry, South China Agricultural University, Guangzhou, 510642 Guangdong P. R. China; 2 Guangdong provincial key laboratory for the development biology and environmental adaptation of agricultural organisms, College of Life Sciences, South China Agricultural University, Guangzhou, 510642 Guangdong P. R. China; 3 Guangdong-Hong Kong-Macao Joint Laboratory for Contaminants Exposure and Health, School of Public Health, Guangdong Pharmaceutical University, Guangzhou, 510006 P. R. China

**Keywords:** Gut microbiome, Antibiotics, Dietary fiber, American gut project, Bifidobacterium, Precision nutrition

## Abstract

**Aim:**

Dietary fiber is a key modulator of the gut microbiome, yet its specific role following antibiotic exposure remains under-characterized in large populations. Previous studies suggest high-fiber diets promote recovery, but often rely on small cohorts. We aimed to re-evaluate these microbial signatures and their association with current microbiome states in a large, diverse adult population.

**Methods:**

We analyzed 16 S rRNA gene sequencing data from the American Gut Project (AGP). Participants with recent antibiotic exposure were stratified by high-fiber (HF; *N* = 971) or low-fiber (LF; *N* = 955) intake. We assessed alpha and beta diversity and identified differentially abundant genera using LEfSe. Key biomarkers were validated using ANCOM-BC and multivariable linear regression adjusting for age, sex, and BMI.

**Results:**

Contrary to previous models, high-fiber intake was not associated with a uniform enrichment of commensal Clostridia. Instead, *Bifidobacterium* and *Lachnospira* were identified as genus-level biomarkers significantly enriched in the HF group, while *Bacteroides* and *Parabacteroides* were enriched in the LF group. These associations were confirmed to be robust by multivariable linear regression (*P* < 0.001). High-fiber intake was not associated with significantly higher alpha diversity within the one-month post-antibiotic timeframe.

**Conclusion:**

Post-antibiotic microbiome signatures associated with fiber intake are distinct and specific. We identified *Bifidobacterium* and *Lachnospira* as robust targets for dietary interventions, challenging simplistic models of recovery and highlighting the need for precision nutrition strategies to enhance gut resilience.

**Supplementary Information:**

The online version contains supplementary material available at 10.1186/s13104-026-07708-7.

## Introduction

Antibiotic usage is a primary driver of gut microbiome dysbiosis and the proliferation of antimicrobial resistance genes, posing a global public health challenge [1]. Consequently, antibiotic exposure profoundly disrupts the gut microbiome, making its rapid recovery essential for restoring digestive function, nutrient metabolism, and colonization resistance against opportunistic pathogens like *Clostridioides difficile* infection (CDI) [2, 3]. Dietary fiber is recognized as a key modulator of this recovery process. Several studies suggest that high-fiber diets post-antibiotics promote beneficial taxa like commensal Clostridia (primarily members of clusters IV and XIVa) and Bacteroidetes, while low-fiber conditions may foster facultative anaerobes (e.g., *Enterococcus*, *Streptococcus*, *Klebsiella*) [4, 5]. However, the validation of these proposed biomarkers has been limited. Studies involving antibiotic administration to human subjects often face constraints on sample size due to ethical and logistical considerations. For instance, the Tanes et al. study involved 30 human subjects across three dietary arms in an interventional design [4], while Hewlett et al.‘s human evidence was derived from an even smaller cohort, with key microbial insights largely reliant on their murine model [5]. We aimed to re-evaluate and refine these patterns in a significantly larger adult population using data from the American Gut Project (AGP) [6].

## Methods

AGP is the largest publicly available human microbiome dataset, which includes antibiotic history and dietary frequency information, allowing for a robust assessment of post-antibiotic dietary fiber impact in a broad adult population. We utilized publicly available 16 S rRNA gene V4 region sequencing data and associated lifestyle/dietary metadata from AGP, obtained from the project’s public FTP repository (http://ftp.microbio.me/AmericanGut/latest/) [6]. For our analyses, we used genus-level feature tables, which represent Amplicon Sequence Variants (ASVs) aggregated to the genus level. These tables were generated by the AGP/QIITA (Qiita ID: 10317) platform employing their standard processing protocols, which include sequence denoising and ASV generation using DADA2 within the QIIME 2 framework [7, 8]. The use of this pre-processed table ensures consistency with the standardized AGP pipeline. As part of this protocol, taxonomic assignments for these ASVs were performed against the Greengenes database (version 13_8) [9].

Only adult participants (≥ 18 years) were included. Antibiotic exposure was defined as “ABX_Recent” (antibiotics in the last week/month) or “ABX_None” (no antibiotics in the past year). A composite dietary fiber score (0–12 points based on fruit, vegetable, and whole grain intake frequency; Never = 0, Rarely (a few times/month) = 1, Occasionally (1–2 times/week) = 2, Regularly (3–5 times/week) = 3, Daily = 4) was used to categorize participants into Low Fiber (LF; score ≤ 4) or High Fiber (HF; score ≥ 9). These thresholds were chosen to create a distinct contrast between participants with consistently low versus high fiber consumption patterns, thereby minimizing the inclusion of individuals with intermediate intake and maximizing the potential to detect a biological signal. The LF cutoff (≤ 4) generally corresponds to individuals consuming key fiber sources “Rarely” to “Occasionally”, while the HF cutoff (≥ 9) corresponds to individuals consuming them “Regularly” to “Daily”. This resulted in four primary comparison groups: ABX_Recent + LF (*N* = 955), ABX_Recent + HF (*N* = 971), ABX_None + LF (*N* = 2,679), and ABX_None + HF (*N* = 2,835). Demographic and baseline characteristics, including age, sex, BMI, race/ethnicity, education level, were largely comparable across these groups (Table 1), minimizing confounding effects in our primary analyses.

**Table 1:** Demographic and baseline characteristics of American gut project (AGP) adult participant groups

Characteristic	ABX_Recent + LF (*N* = 955)	ABX_Recent + HF (*N* = 971)	ABX_None + LF (*N* = 2679)	ABX_None + HF (*N* = 2835)	*P*-value
age	48 [36,61]	51 [37,64]	47 [36.59]	50 [37,61]	0.06
male	43.04%	40.27%	45.43%	43.81%	0.12
BMI	23.34 [21.08,26.57]	23.43 [21.35,26.61]	23.38 [21.1,26.22]	23.10 [21.21,25.77]	0.85
Minority	10.68%	10.81%	10.01%	10.93%	0.67
Education level	3 [3,4]	4 [3,4]	4 [3,4]	4 [3,4]	0.23

Data are presented as Median [Interquartile Range (IQR)] for continuous variables (age, BMI) and percentage (%) for categorical variables (male, minority). Education level is presented as Median [IQR] based on self-reported categories numerically coded as follows: 0="Did not complete high school”, 1="High School or GED equivalent”, 2="Some college or technical school”, 3="Associate’s degree”, 4="Bachelor’s degree”, 5="Some graduate school or professional”, 6="Graduate or Professional degree”. Groups are defined by recent antibiotic use (“ABX_Recent”: antibiotics in the last week/month; “ABX_None”: no antibiotics in the past year) and a composite dietary fiber score (“LF” Low Fiber: score ≤ 4; “HF” High Fiber: score ≥ 9). P-values for comparisons across the four groups were determined using Kruskal-Wallis test for continuous/ordinal variables and Chi-squared test for categorical variables

All statistical analyses were performed in R (version 4.2.1). The R scripts used to perform the data analysis and generate the figures for this study are available in the Supplementary Methods file. The genus-level feature table containing raw sequence counts was used as the starting point. Alpha diversity was calculated using the ‘Observed Features’ metric on the raw counts and was compared across the four primary groups using a Kruskal-Wallis test. For beta diversity and differential abundance analyses, the raw count data was normalized to relative abundances by Total Sum Scaling (TSS). Principal Coordinates Analysis (PCoA) based on Bray-Curtis dissimilarity was used to visualize community structure differences. The statistical significance of group clustering was assessed using a Permutational Multivariate Analysis of Variance (PERMANOVA) with 999 permutations, as implemented in the vegan package. To account for potential confounding effects, age and BMI were included as covariates in the model. Differentially abundant taxa between groups were identified using LEfSe (Linear Discriminant Analysis Effect Size). This analysis was performed on the relative abundance table, and we considered taxa with a Kruskal-Wallis p-value < 0.05 and a Linear Discriminant Analysis (LDA) score > |2.0| as significant biomarkers. To test the robustness of the key biomarkers identified by LEfSe against potential confounding, we performed a sensitivity analysis using multivariable linear regression within the ABX_Recent group, in which the log-transformed relative abundance of each key genus was modeled against the continuous dietary fiber score while adjusting for age, sex, and BMI.

To validate the biomarkers identified by LEfSe using a compositionally-aware framework, we employed ANCOM-BC [10]. For this analysis, we utilized the raw ASV count data extracted from the original AGP BIOM file, as ANCOM-BC requires integer counts. ASV counts were aggregated to the genus level. The model was fitted to the ABX_Recent subset to test for the effect of fiber group (HF vs. LF), adjusting for age, sex, and BMI as covariates. Significance was defined as an FDR-adjusted p-value (q-value) < 0.05.

Finally, to test the robustness of our key biomarker findings, a sensitivity analysis was performed using multiple linear regression. For each of the four main genera (*Bifidobacterium*, *Lachnospira*, *Bacteroides*, *Parabacteroides*), we modeled their log10-transformed relative abundance (log10[relative abundance + 1e-5]) as the dependent variable. The continuous dietary fiber score (0–12) was used as the primary independent variable, with age, sex, and BMI included as covariates in all models.

## Results

**Fig. 1:**
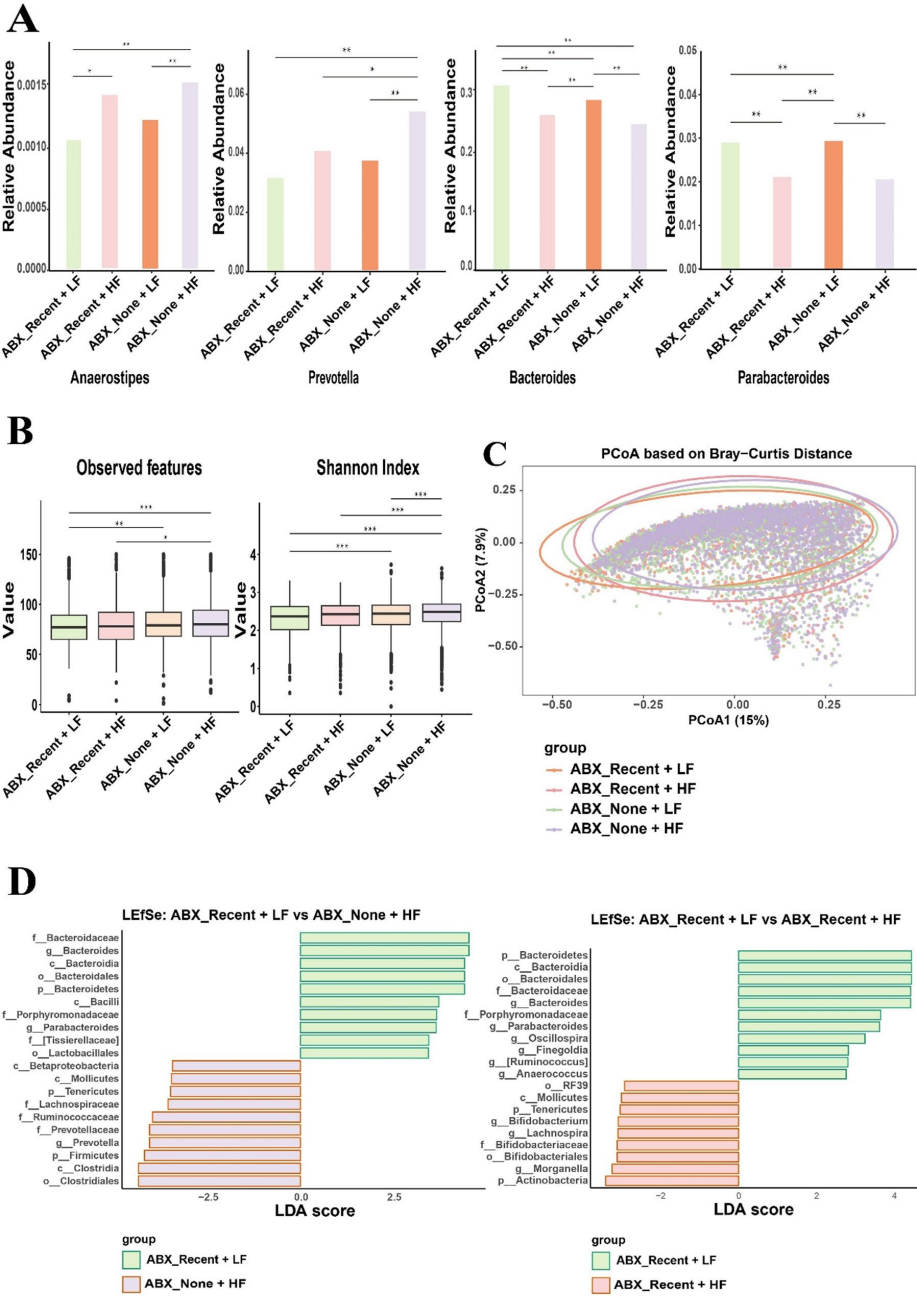
Gut Microbiome Signatures Associated with Dietary Fiber Intake Following Recent Antibiotic Exposure in a Large Adult Cohort from the American Gut Project. **A** Relative abundance of selected bacterial genera across four defined participant groups. Groups are: ABX_Recent + LF (recent antibiotic use, low fiber intake; *N* = 955), ABX_Recent + HF (recent antibiotic use, high fiber intake; *N* = 971), ABX_None + LF (no recent antibiotic use, low fiber intake; *N* = 2,679), and ABX_None + HF (no recent antibiotic use, high fiber intake; *N* = 2,835). Bar plots represent the mean relative abundance. Significant differences between groups were determined by Kruskal-Wallis test. **B** Alpha diversity metrics across the four participant groups. The box represents the interquartile range (IQR), the horizontal line within the box indicates the median, and whiskers extend to 1.5 times the IQR. Outliers are shown as individual points. Alpha diversity was calculated for each participant and compared across groups using Kruskal-Wallis tests. *p < 0.05, **p < 0.01, ***p < 0.001. **C** Beta diversity visualized by Principal Coordinates Analysis (PCoA) based on Bray-Curtis distances. Each point represents the microbiome profile of an individual participant, colored by their respective group as indicated in the legend. Ellipses represent 95% confidence intervals for each group. Overall group differences in community structure were assessed using Permutational Multivariate Analysis of Variance (PERMANOVA) with 999 permutations on the Bray-Curtis distance matrix (Overall R²=0.01, *p* < 0.001). **D** Differentially abundant taxa identified by Linear Discriminant Analysis Effect Size (LEfSe). Left panel: Biomarkers distinguishing ABX_Recent + LF from ABX_None + HF (representing a “healthy, high-fiber baseline”). Right panel: Biomarkers distinguishing ABX_Recent + LF from ABX_Recent + HF. Only taxa meeting an LDA score threshold >|2.0| and a p-value < 0.05 are shown. Bars indicate the effect size (LDA score) for each taxon. Taxonomic levels are indicated (p_ Phylum, c_ Class, o_ Order, f_ Family, g_ Genus). Bars indicate the effect size (LDA score) for each taxon

Contrary to expectations derived from previous models, we found that the association between the Class Clostridia and high-fiber intake was not uniform at the genus level. Only *Anaerostipes* showed a significantly higher relative abundance with HF diets, irrespective of recent antibiotic use (Fig. 1A). Other prominent Clostridia genera, such as *Faecalibacterium*, *Roseburia*, and *Blautia*, did not consistently associate with HF status in our cohort (Supplementary Figure S1). Surprisingly, *Eubacterium* exhibited higher abundance in LF groups, both with and without recent antibiotic exposure (Supplementary Figure S1). Within Bacteroidetes, *Prevotella* abundance was higher in HF groups, consistent with previous findings. However, *Bacteroides* and *Parabacteroides* were more abundant in LF groups, regardless of antibiotic history (Fig. 1A). Furthermore, the anticipated increase in facultative anaerobes like *Enterococcus*, *Streptococcus*, or *Klebsiella* in LF groups was not observed (Supplementary Figure S1), suggesting that in a general adult population, a simple reduction in dietary fiber post-antibiotics may not universally trigger a pronounced bloom of these specific opportunistic taxa. These results suggest that while broad taxonomic shifts may occur, considerable intra-group variation exists; simple phylum or class-level associations with fiber intake post-antibiotics may be overly simplistic in large human populations.

Microbial alpha diversity was significantly reduced in ABX_Recent groups compared to ABX_None groups (Fig. 1B; Kruskal-Wallis, *p* < 0.001). An exploratory sub-analysis stratifying the ABX_Recent group by timing of exposure (‘last week’ vs. ‘last month’) did not reveal a statistically significant difference in alpha diversity between these two subgroups (p > 0.05), potentially due to reduced statistical power. Interestingly, while HF was associated with higher alpha diversity in the ABX_None group, this benefit was not apparent in the ABX_Recent group, suggesting that high fiber intake alone may not rapidly restore diversity within one-month post-antibiotics, or that longer recovery periods are needed to observe this effect. Beta diversity analysis (Bray-Curtis, PCoA) revealed small but statistically significant clustering by group (Fig. 1C; PERMANOVA, p < 0.001, R²=0.01). However, the effect size was minimal (R²=0.01), indicating that the combination of recent antibiotic use and dietary fiber intake explains only 1% of the total variance in gut microbial community structure. This suggests that while a discernible pattern exists, inter-individual variation is a far more dominant factor shaping the overall microbiome composition.

LEfSe analysis identified distinct microbial signatures differentiating these groups (Fig. 1D). To first understand the microbial profile of a low-fiber diet following recent antibiotic use relative to a healthy, high-fiber baseline, we compared the ABX_Recent + LF group to the ABX_None + HF group. ABX_None + HF group was notably enriched in taxa generally considered beneficial or indicative of a fiber-utilizing community, including *Prevotella*, Lachnospiraceae, and the Class Clostridia. Conversely, the ABX_Recent + LF group showed an enrichment of *Bacteroides*, *Parabacteroides*, and the class Bacilli and Bacteroidia. Next, to assess the impact of dietary fiber specifically within the post-antibiotic context, we compared the ABX_Recent + LF group to the ABX_Recent + HF group. Several genera including *Bacteroides*, *Parabacteroides*, *Oscillospira*, *Finegoldia*, *Ruminococcus*, and *Anaerococcus* remained enriched in the ABX_Recent + LF group. In contrast, the ABX_Recent + HF group showed a significant enrichment of *Bifidobacterium*, *Lachnospira*, and *Morganella* as biomarkers.

To validate these LEfSe findings with a compositionally-aware method, we performed a confirmatory analysis using ANCOM-BC. The results confirmed our primary findings: *Bifidobacterium* and *Lachnospira* were significantly enriched in the ABX_Recent + HF group, while *Bacteroides* and *Parabacteroides* were significantly enriched in the ABX_Recent + LF group. However, some secondary biomarkers identified by LEfSe (e.g., *Finegoldia*, *Anaerococcus*) did not reach statistical significance in the ANCOM-BC model, suggesting that the core signal of the post-antibiotic fiber response is driven by specific high-abundance taxa (Supplementary Table 1).

Notably, while Class Clostridia was a strong marker for the ABX_None + HF “healthy baseline,” it was not significantly enriched in the ABX_Recent + HF group when compared directly to ABX_Recent + LF. This suggests that while a high-fiber diet post-antibiotics is associated with a significant enrichment of certain beneficial genera like *Bifidobacterium* and some *Lachnospira*, the broader presence of the Class Clostridia to levels seen in an undisturbed high-fiber state may be delayed or contingent on factors beyond general fiber quantity within this timeframe. This could involve the need for more specific fiber types, the influence of microbial niches, cross-feeding interactions, or ‘founder effects’ within the recovering ecosystem playing a dominant role in the re-establishment of many Clostridia members [11, 12].

To ensure these findings were not an artifact of our dichotomized fiber grouping, we performed a sensitivity analysis using the continuous fiber score (0–12) as a predictor. This analysis was performed on the ABX_Recent group and adjusted for age, sex, and BMI. The results strongly confirmed our primary findings: the continuous fiber score was a significant predictor for the log-transformed abundance of all key genera. Specifically, it was positively and significantly associated with *Bifidobacterium* (β = 0.049, *p* < 0.001) and *Lachnospira* (β = 0.027, *p* < 0.001), and negatively and significantly associated with *Bacteroides* (β = -0.009, *p* < 0.001) and *Parabacteroides* (β = -0.014, *p* < 0.001). This confirms the robustness of these associations and indicates a dose-response relationship between fiber intake and the abundance of these key post-antibiotic biomarkers (Table 2).

**Table 2:** Association of continuous dietary fiber score with key genera in the ABX_Recent group

Dependent variable (Genus)	Independent variable	Estimate (β)	95% Confidence interval	*P*-value
*Bifidobacterium*	Fiber Score	0.049	(0.040, 0.057)	< 0.001
*Lachnospira*	Fiber Score	0.027	(0.021, 0.033)	< 0.001
*Bacteroides*	Fiber Score	-0.009	(-0.013, -0.006)	< 0.001
*Parabacteroides*	Fiber Score	-0.014	(-0.019, -0.008)	< 0.001

The table displays the coefficient (Estimate), 95% Confidence Interval, and significance (P-value) for the Fiber_Score term. All models were adjusted for age, sex, and BMI. The dependent variable was the log10-transformed relative abundance of each genus

## Discussion

A key strength of our study is the utilization of the large and diverse AGP cohort, significantly increasing the sample size and ethnic/geographic diversity compared to previous investigations, thus enhancing the generalizability of our findings regarding post-antibiotic dietary responses in adults. The standardized data processing through QIITA also ensures methodological consistency.

Our study has limitations inherent to the cross-sectional nature of the AGP dataset. First, as participants were sampled at a single time point, our analyses reveal associations with post-antibiotic states rather than temporal recovery dynamics. We acknowledge that terms like ‘restoration’ imply a process we cannot directly observe; thus, our findings should be interpreted as distinct microbial profiles associated with dietary habits in a post-antibiotic context. Second, the metrics for our key variables—dietary fiber intake and antibiotic exposure—were coarse. The dietary fiber score, while practical, lacks precision regarding specific fiber types (e.g., resistant starch) and quantities. Similarly, the ‘recent antibiotic’ category obscures critical heterogeneity regarding drug class, dosage, and duration. Different antibiotics have vastly different spectra of activity and therefore exert distinct selective pressures on the gut microbiota. This significant unmeasured heterogeneity within the ABX_Recent group could mask more nuanced, drug-specific associations and is a key constraint on the biological interpretability of our findings. It is plausible that the specific class of antibiotic has a substantially larger effect on microbial composition than dietary fiber, representing a major unmeasured confounder in our study. Third, the AGP cohort is subject to self-selection bias, potentially limiting generalizability to less health-conscious or clinical populations. Fourth, regarding statistical limitations, the primary LEfSe analysis does not adjust for potential confounders. To address this, we validated our key biomarker findings using the compositionally-aware method ANCOM-BC and multivariable linear regression, which confirmed the robustness of our results. While we confirmed these key variables were largely balanced across our study groups, we cannot rule out the possibility of residual confounding influencing the identified associations. Specifically, the AGP dataset does not contain information on factors such as concurrent probiotic use or detailed regional dietary patterns, which could also influence the microbiome. Therefore, the genus-level biomarkers reported here should be considered preliminary until they are validated using covariate-adjusted models in future studies. Fifth, our analytical approach also has inherent limitations related to taxonomic resolution. We relied on taxonomic assignments performed with the Greengenes 13_8 database, as this was the standard used in the pre-processed AGP/QIITA data we analyzed. We acknowledge that this database may lack the accuracy and completeness of more modern databases like SILVA or GTDB, which could impact the precise assignment of some taxa, particularly within taxonomically complex and frequently reclassified families such as the Lachnospiraceae and Eubacteriaceae, to which some of our identified genera belong. Furthermore, our decision to analyze data aggregated at the genus level, while improving statistical power and enabling comparison with previous literature, necessarily obscures potential species- and strain-level heterogeneity. Different species within a single genus can have distinct or even opposing functional roles. We acknowledge that describing entire genera with broad functional labels like ‘beneficial’ or ‘opportunistic’ is a simplification, as the true ecological and functional capacity is often highly specific to the species or even strain level. Therefore, our genus-level findings represent broad associations that require further investigation with higher-resolution methods, such as shotgun metagenomics, to confirm and dissect the specific functional contributions of the underlying species.

Beyond these associations, our findings invite speculation on the underlying ecological mechanisms driving these distinct microbial signatures. The enrichment of *Bifidobacterium* in the high-fiber (HF) group is consistent with its established role as a primary degrader of complex plant-derived carbohydrates, particularly prebiotic fructans like inulin and galactooligosaccharides (GOS), which are often inaccessible to other gut microbes [13, 14]. By initiating the breakdown of these fibers, *Bifidobacterium* may engage in cross-feeding, producing metabolites that support the growth of other beneficial taxa. The concurrent enrichment of *Lachnospira*, a well-known butyrate producer [15], supports this view. Butyrate is a critical short-chain fatty acid that serves as the primary energy source for colonocytes and enhances gut barrier function, a process particularly relevant for gut homeostasis after antibiotic-induced disruption [16–18]. Conversely, the significant enrichment of *Bacteroides* and *Parabacteroides* in the low-fiber (LF) group may reflect their notable metabolic flexibility. In an environment scarce in dietary fiber, certain species within these genera are known to be capable of switching their metabolism to utilize host-derived glycans from the colonic mucus layer [19, 20]. A bloom of these mucin-degrading bacteria in a low-fiber, post-antibiotic setting could therefore have important implications for gut barrier integrity and warrants further investigation.

A noteworthy finding was the lack of a uniform enrichment of many prominent commensal Clostridia genera (such as *Faecalibacterium* and *Roseburia*) in the high-fiber group post-antibiotics, which contrasts with some models of fiber-mediated recovery. This suggests a more complex ecological dynamic than simple dietary substrate availability. One explanation could be delayed recolonization; many Clostridia are spore-formers that, while resilient, may exhibit slower germination and outgrowth kinetics compared to non-spore-forming primary colonizers [21], meaning their recovery may extend beyond the one-month timeframe of our study. Alternatively, ‘founder effects’ and competitive exclusion could play a significant role in the sparsely populated post-antibiotic gut, where fast-growing primary fiber-degraders like *Bifidobacterium* might rapidly occupy niches and consume available resources, thereby impeding the immediate re-establishment of certain Clostridia [22]. Finally, the effect may be substrate-specific; a general high-fiber diet may not necessarily be enriched in the particular prebiotic fibers, such as pectin or inulin, that are preferentially utilized by key Clostridia like *Faecalibacterium* [23, 24]. These possibilities highlight that restoring the full diversity of the fiber-degrading community is a complex process contingent on ecological priority effects and specific substrate availability.

In conclusion, our analysis of a large adult cohort provides a more nuanced view of microbiome responses to dietary fiber following antibiotic use. While some previously suggested patterns (e.g., *Prevotella* and HF) hold, others (e.g., a uniform increase of ‘commensal Clostridia’ or a clear bloom of specific facultative anaerobes with LF) are not straightforwardly generalizable. We identify novel genus-level biomarkers, such as the enrichment of *Bifidobacterium* and specific *Lachnospira* species with high fiber intake post-antibiotics, versus *Bacteroides* and *Parabacteroides* with low fiber. These findings underscore the complexity of gut microbiome response after antibiotic exposure and highlight the urgent need for more personalized and targeted dietary strategies (e.g., specific fibers or prebiotics/synbiotics). Specifically, our identification of *Bifidobacterium* and *Lachnospira* as robust responders to high fiber intake post-antibiotics suggests that future longitudinal intervention studies could focus on prebiotic fibers known to selectively promote these genera, such as inulin or galactooligosaccharides (GOS), to test their efficacy in enhancing gut resilience. The enrichment of *Lachnospira* in the high-fiber group is consistent with strategies aiming to restore colonization resistance via short-chain fatty acids, as previous work has demonstrated that modulating the gut microbiota to enhance butyrate production can offer protection against environmental stressors [25]. This work highlights the necessity of validating findings in large, diverse populations to guide precise interventions for enhancing gut resilience and potentially reducing CDI risk. Ultimately, understanding the interplay between diet and antibiotics is part of a broader scientific imperative to predict and manage the health consequences of ecosystems responding to acute environmental stressors, whether in the human gut or on a global scale [26].

## Supplementary Information

Below is the link to the electronic supplementary material.

Supplementary Material 1.

Supplementary Material 2.

Supplementary Material 3.

## Data Availability

The dataset analyzed during the current study is publicly available in the American Gut Project FTP repository, http://ftp.microbio.me/AmericanGut/latest/.
