# Supplementary material for: Re-evaluating gut microbiome signatures of post-antibiotic dietary fiber intake in a large adult cohort
Source: BMC Res Notes. 2026 Feb 14;19:124. doi: 10.1186/s13104-026-07708-7 (PMC13011435; doi:10.1186/s13104-026-07708-7)
Supplement: Supplementary file 1 — Supplementary Material 1. [file 13104_2026_7708_MOESM1_ESM.docx]

R Code for Statistical Analysis and Visualization This document provides the R code used for the primary statistical analyses and figure generation in the manuscript "Re-evaluating Gut Microbiome Signatures of Post-Antibiotic Dietary Fiber Intake in a Large Adult Cohort." All analyses were performed in R version 4.2.1.

A：

library(ggplot2)

library(dplyr)

library(openxlsx)

data <- read.xlsx("Bacteroides.xlsx")

data$group <- stringr::str_split(data$group,"-",simplify = T)[,1]

data$group <- factor(data$group,levels = c("ABX_Recent + LF","ABX_Recent + HF","ABX_None + LF","ABX_None + HF"))

str(data$expression)

data$expression <- as.numeric(trimws(as.character(data$expression)))

sum(is.na(data$expression))

data$expression[is.na(data$expression)] <- 0

sum_data <- data %>%

group_by(gene, group) %>%

summarise(

expression_mean = mean(expression, na.rm = TRUE),

expression_sd = sd(expression, na.rm = TRUE),

.groups = 'drop'

)

head(sum_data)

Bacteroides_data <- sum_data %>% filter(gene == "Bacteroides")

ggplot(Bacteroides_data, aes(x = group, y = expression_mean, fill = group)) +

geom_bar(

stat = "identity",

width = 0.3,

color = "white"

) +

scale_fill_manual(values = my_colors) +

scale_y_continuous(

expand = expansion(mult = c(0, 0.1)),

limits = c(0, NA)

) +

theme_classic(base_size = 14) +

theme(

legend.position = "right",

axis.text.x = element_text(

angle = 45,

hjust = 1,

vjust = 1

),

axis.line = element_line(color = "black"),

panel.background = element_blank()

) +

labs(x = "Bacteroides", y = "Relative Abundance")

## 定义差异分析函数

func_HSD <- function(data,formula){

aov_re <- aov(formula,data)

hsd_re <- TukeyHSD(aov_re)

pvalue <- as.data.frame(hsd_re$group)

return(pvalue)

}

formula <- expression~group

pvalue <- func_HSD(data,formula)

pvalue$aster <- ifelse(pvalue$`p adj`<= 0.01,"**",

ifelse(pvalue$`p adj`<= 0.05,"*","NS"))

View(pvalue)

B：

library(openxlsx)

library(ggplot2)

d1 <- read.xlsx("observed.xlsx", sep = "\t")

d1$region <- factor(d1$region, levels = c("ABX_Recent + LF","ABX_Recent + HF","ABX_None + LF","ABX_None + HF"))

yanse <- c( "#E2F2CD", "#F9D5D5",'#FBE3CD', "#E8E0EF")

p <- ggplot(d1, aes(x = region, y = observed, fill = region)) +

scale_fill_manual(values = yanse) +

geom_boxplot() +

theme_minimal() + # 设置背景为白色

theme(legend.position = "right") +

theme(axis.text.x = element_text(size = 16, color = "black", angle = 90, hjust = 1, vjust = 0.5),

axis.text.y = element_text(size = 16, color = "black")) +

theme(panel.grid = element_blank()) + # 隐藏网格线

theme(axis.line = element_line(color = "black", linewidth = 0.5),

legend.title = element_text(size = 16),

legend.text = element_text(size = 16)) + # 显示坐标轴线

theme(axis.ticks = element_line(color = "black", linewidth = 0.5),

text = element_text(size = 14),

legend.key.size = unit(1, 'cm')) + # 显示刻度线

labs(x = "Region", y = "Observed features") + # 设置坐标轴标签

scale_y_continuous(limits = c(0,200 )) # 设置 y 轴范围

print(p)

ggsave("observed.pdf", plot = p, width = 15, height = 12, units = "in", dpi = 300)

library(FSA)

kruskal_result <- kruskal.test(observed ~ region, data = d1)

print(kruskal_result)

# Dunn 检验

dunn_result <- dunnTest(observed ~ region, data = d1, method = "bonferroni")

print(dunn_result)

# 提取显著性差异的组对

significant_comparisons1 <- dunn_result$res[dunn_result$res$P.adj < 0.05, ]

write.xlsx(significant_comparisons1,file="observed_sig.xlsx")

C:

library(vegan)

library(ggplot2)

library(ape)

library(dplyr)

otu <- read.xlsx("otu_table1.xlsx")

rownames(otu) <- otu[, 1]

otu <- otu[, -1]

metadata <- read.xlsx("metadata.xlsx")

bray_dist <- vegdist(otu, method = "bray")

pcoa_result <- cmdscale(bray_dist, eig=TRUE, k=3)

pcoa_scores <- as.data.frame(pcoa_result$points)

colnames(pcoa_scores) <- paste0("PCoA", 1:ncol(pcoa_scores))

pcoa_scores$Group <- metadata$Group

variance_exp <- round(pcoa_result$eig / sum(pcoa_result$eig[pcoa_result$eig>0]) * 100, 1)

adonis_result <- adonis2(bray_dist ~ Group, data=metadata, permutations=999)

print(adonis_result)

write.xlsx(adonis_result,file="adonis_result.xlsx")

ggplot(pcoa_scores, aes(x = PCoA1, y = PCoA2, color = Group)) +

geom_point(size = 1, alpha = 0.8) + # 减小点大小（原3→2）

stat_ellipse(level = 0.95, linewidth = 0.9, linetype = "solid") +

labs(x = paste0("PCoA1 (", variance_exp[1], "%)"),

y = paste0("PCoA2 (", variance_exp[2], "%)"),

title = "PCoA based on Bray-Curtis Distance") +

scale_color_manual(values = c("#F49568", "#EF98A1","#B6DAA7","#C2B1D7")) +

theme_bw() +

theme(panel.grid = element_blank(),

legend.position = "right",

plot.title = element_text(hjust = 0.5))

D：

library(tidyverse)

library(microeco)

library(magrittr)

library(patchwork)

otu <- read.xlsx("otu.xlsx")

rownames(otu) <- otu[, 1]

otu <- otu[, -1]

group <- read.xlsx("group.xlsx")

rownames(group) <- group[, 1]

tax <- read.xlsx("tax1.xlsx")

rownames(tax) <- tax[, 1]

tax <- tax[, -1]

dataset <- microtable$new(sample_table = group,

otu_table = otu,

tax_table = tax)

dataset

target_groups <- c("group1", "group4")

filtered_dataset <- dataset$clone()

filtered_samples <- dataset$sample_table[dataset$sample_table$group %in% target_groups, ]

filtered_dataset$sample_table <- filtered_samples

filtered_dataset$otu_table <- dataset$otu_table[, rownames(filtered_samples)]

table(filtered_dataset$sample_table$group)

filtered_dataset$cal_abund()

# 运行LEfSe分析

lefse_result <- trans_diff$new(

dataset = filtered_dataset,

method = "lefse",

group = "group", # 分组列名

alpha = 0.05, # p值阈值

lefse_subgroup = NULL # 无亚组分析

)

head(lefse_result$res_diff)

my_colors <- c("group1" = "#F49568" , "group4" ="#F9D5D5")

p_bar <- lefse_result$plot_diff_bar(

use_number = 1:20,

width = 0.8,

group_order = names(my_colors)

) +

scale_fill_manual(values = my_colors) +

labs(title = "LEfSe: ABX_Recent + LF vs ABX_None + HF") +

theme_minimal() +

theme(

panel.grid.major = element_blank(),

panel.grid.minor = element_blank(),

axis.line = element_line(color = "black")

)

print(p_bar)
