# Supplementary material for: Re-evaluating gut microbiome signatures of post-antibiotic dietary fiber intake in a large adult cohort
Source: BMC Res Notes. 2026 Feb 14;19:124. doi: 10.1186/s13104-026-07708-7 (PMC13011435; doi:10.1186/s13104-026-07708-7)
Supplement: Supplementary file 2 — Supplementary Material 2. [file 13104_2026_7708_MOESM2_ESM.docx]

**
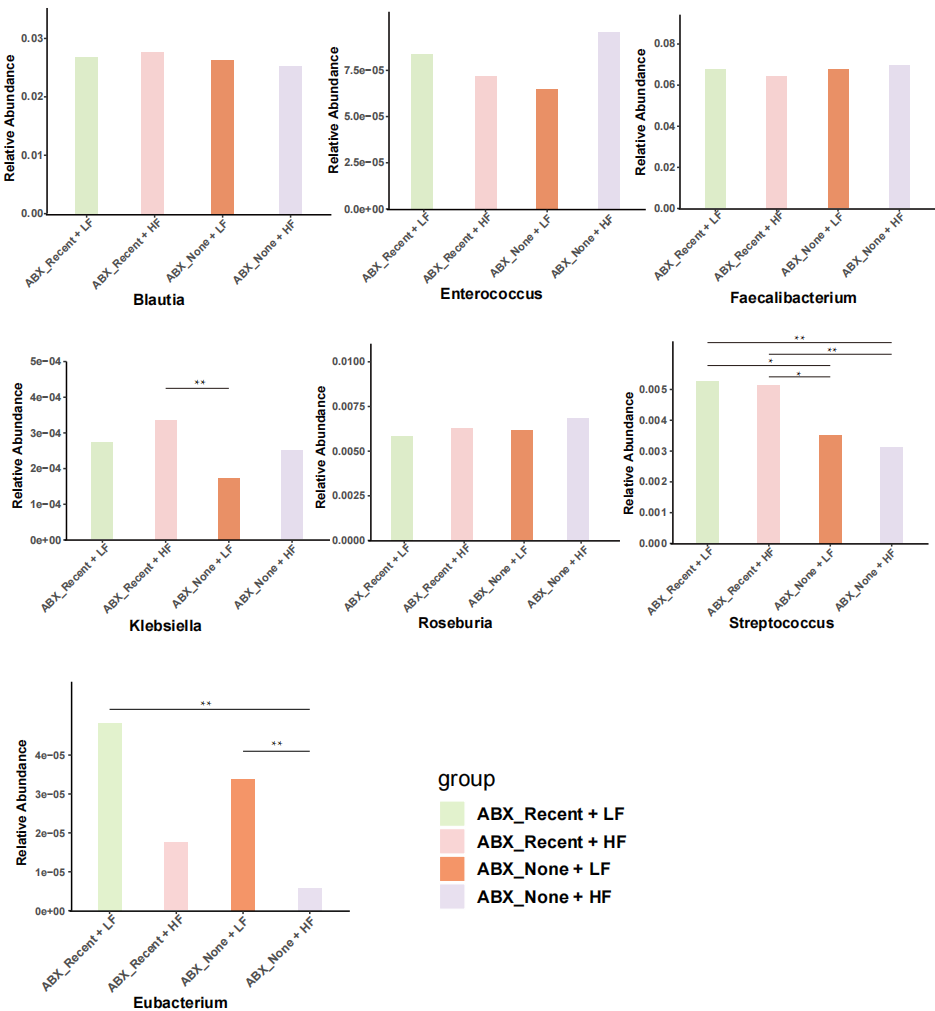
**

Supplementary Figure 1. Relative Abundance of Selected Bacterial Genera Implicated in Previous Studies Across Defined Participant Groups. Bar plots show the mean relative abundance of (A) *Blautia*, (B) *Enterococcus*, (C) *Faecalibacterium*, (D) *Klebsiella*, (E) *Roseburia*, (F) *Streptococcus*, and (G) *Eubacterium* across four participant groups: ABX_Recent + LF (recent antibiotic use, low fiber intake; N=955), ABX_Recent + HF (recent antibiotic use, high fiber intake; N=971), ABX_None + LF (no recent antibiotic use, low fiber intake; N=2,679), and ABX_None + HF (no recent antibiotic use, high fiber intake; N=2,835). Significant differences between groups were determined by Kruskal-Wallis test. * p < 0.05, * p < 0.01.
